# Toxic Air Contaminant Emissions from Residential and Commercial Fossil Gas Appliances: A Systematic Review

**DOI:** 10.1007/s40572-026-00562-6

**Published:** 2026-09-16

**Authors:** Erika Tam, Jeremy K. Domen, Drew R. Michanowicz, Misbath Daouda

**Affiliations:** 1 School of Public Health, University of California, Berkeley, Berkeley, CA USA; 2 PSE Healthy Energy, Oakland, CA USA

**Keywords:** Toxic air contaminants, Gas appliances, Emission factors, Appliance standards

## Abstract

**Purpose of Review:**

Gas appliances are increasingly recognized as sources of indoor toxic air contaminant (TAC) exposure, yet no prior review has systematically synthesized emission factor (EF) data across appliance types and fuels. We reviewed the literature to characterize TAC emissions from residential and commercial gas appliances, identify data gaps, and assess implications for regulation and public health.

**Recent Findings:**

Twenty-five studies met inclusion criteria. Evidence of nonzero emissions was identified for at least 41 TACs released through gas leakage and combustion pathways. Formaldehyde and benzene were the only compounds with sufficient data for quantitative characterization, with EFs varying by up to two orders of magnitude across studies and operating conditions. The literature was heavily concentrated on cooking stoves, while heaters, water heaters, and dryers were rarely studied. Emerging evidence suggests methane and carbon monoxide measurements may support inference-based estimation of leakage- and combustion-related TAC emissions.

**Summary:**

Gas appliances emit multiple carcinogenic and respiratory toxicants through both leakage and combustion. Expanded monitoring, standardized EF methodologies, and targeted measurement campaigns for understudied appliances are needed to support appliance standards, emissions inventories, and health-protective building decarbonization policies.

**Supplementary Information:**

The online version contains supplementary material available at https://doi.org/10.1007/s40572-026-00562-6.

## Introduction

Natural gas has become the dominant energy source in residential and commercial buildings in the United States (U.S.), supplying energy to approximately 73 million households, or nearly half of all homes, for space heating, water heating, cooking, and clothes drying. [1–3] The national gas distribution network spans more than 2.1 million miles of transmission and distribution pipelines, and the number of residential gas customers has grown steadily over the past decade. [3–5] Despite this prevalence, the health implications of indoor gas combustion have received much less regulatory scrutiny relative to outdoor air pollution from the same fuels. Evidence increasingly shows that residential gas appliances are a significant and underregulated source of indoor toxic air contaminant (TAC) exposure. [6–8]

Exposure to TACs has substantial public health implications. Epidemiological evidence links residential gas cooking to increased odds of childhood asthma and wheeze. [9] Formaldehyde and benzene are both classified as Group 1 human carcinogens by the International Agency for Research on Cancer (IARC). [10] Other combustion-derived TACs including acrolein, acetaldehyde, and polycyclic aromatic hydrocarbons (PAHs) are well known respiratory hazards with varying carcinogenic classifications. [10–12] Environmental justice dimensions amplify these concerns: low-income households, renters, and communities of color are disproportionately likely to live in smaller homes with older, less-efficient gas appliances, and limited kitchen ventilation, [13–16] as well as restricted ability to transition to electric alternatives. [17,18] These factors can create systematic disparities in TAC exposure burdens as is the case for nitrogen dioxide exposure attributable to gas and propane stoves. [19]

These concerns have catalyzed regulatory action across multiple jurisdictions. For example, California’s Air Resources Board (CARB) has identified TAC emissions from gas appliances as a priority within its appliance regulatory program. [20] Achieving health-protective, enforceable standards requires quantitative emission factor (EF) benchmarks against which zero-emission alternative technologies can be evaluated, and against which exposure models and health risk assessments for overburdened communities can be developed. Yet despite the regulatory momentum, no prior systematic synthesis had compiled TAC EF data across residential and commercial gas appliances. Existing reviews have addressed specific pollutants or appliance types, or have relied on outdated and geographically limited datasets. This gap has forced regulators to rely on data that may not be representative and has left the evidence needed to set TAC standards incomplete and fragmented.

Gas-fired appliances emit TACs through three primary pathways; indirectly through natural gas leakage, and directly through both incomplete combustion and chemical reactions. Since delivered natural gas contains trace concentrations of benzene, toluene, ethylbenzene, and xylenes (BTEX), sustained pneumatic leakage can result in elevated chronic indoor exposures. [21–24] Incomplete combustion occurs when oxygen is limited or flame conditions prevent full oxidation, producing formaldehyde, acetaldehyde, acrolein, and other carbonyls even in well-maintained appliances. [25] The magnitude of the resulting concentrations can depend on burner design, air-to-fuel ratio, and ventilation. Third, high temperature kinetic formation pathways can generate secondary pollutants such as benzene and toluene through pyrolytic reactions during combustion, regardless of combustion efficiency. [7] In addition to these pollutants, gas appliances may produce other hazardous compounds that have been less extensively characterized.

To address this critical gap, we conducted a systematic literature review of TAC EFs from residential and commercial gas appliances, including stoves, ovens, water heaters, heaters, and dryers. Our analysis aimed to integrate both measurement-based and modeled EF estimates, identify key data limitations, and consolidate the current state of knowledge. This synthesis provides a scientific foundation to support states and jurisdictions in developing appliance emission standards and advancing building decarbonization efforts that safeguard both climate and public health.

## Methods

### Search Strategy

We searched the Web of Science database for literature related to the emissions of methane, TACs, or hazardous air pollutants (HAPs) from appliances that use natural gas, propane (i.e., liquified petroleum gas (LPG), or biogas. We used the reference list of TACs and HAPs from the CARB to inform our search term strategy. The final search terms can be found in the Supplemental Material (Table S1). Search results were constrained to literature published between January 2000 and August 2024.

### Screening and Eligibility

Records were screened sequentially at the title and abstract stages. Title screening excluded records clearly unrelated to gas appliance emissions. Abstract screening applied four pre-specified inclusion criteria: (1) English language; (2) measurement of HAPs, TACs, or methane emissions; (3) emissions attributable to residential or commercial appliances; and (4) use of natural gas, LPG/propane, or biogas as the fuel source. No geographic restrictions were applied. Studies were excluded if they reported only ambient air quality measurements without appliance-level source apportionment, modeled emission estimates without original measurements, or were secondary reviews without primary data. Full texts passing abstract screening were independently assessed by two reviewers; disagreements were resolved through discussion and, where necessary, by consultation with a third reviewer.

### Data Extraction and Harmonization

From each included study, we extracted EFs for TACs on the CARB designated list along with key study characteristics: fuel type, appliance type, experimental setting (laboratory versus field), geographic location, measurement instrumentation, and combustion test conditions. A single study could contribute multiple EF measurements when EFs were reported for different appliances, pollutants, or experimental conditions; therefore, the number of EF measurements (n) does not correspond to the number of studies (N). Appliance types were standardized using EPA Source Classification Codes to enable consistent cross-study comparison. [26] Where EFs were presented in figures rather than tables, we contacted corresponding authors to request underlying numerical data.

Reported EF units, which varied substantially across studies in both pollutant mass units and fuel quantity metrics, were converted to a common energy-normalized unit of milligrams pollutant per megajoule fuel energy (mg/MJ) using published lower heating values for each fuel type. When study specific conversion factors were not available, we used 46 MJ/kg as the energy density for LPG and 50 MJ/kg or 36.0 MJ/m, [3] for the energy density of natural gas. Once extracted and standardized, EFs were organized by TAC and fuel type. TACs were grouped by frequency of representation in the literature, with categories including benzene, formaldehyde, methane, PAHs, and a residual category of other TACs.

LPG and propane studies were combined into a single LPG category because propane is the main component of LPG and the two fuels have very similar combustion characteristics in residential appliances. Following TAC EF extraction, included studies were additionally screened for the reporting of carbon monoxide (CO) data alongside TAC data to evaluate the feasibility of TAC-CO inference relationships. Quantitative descriptive statistics were computed for TACs with three or more EF measurements; compounds with fewer measurements were documented qualitatively.

## Results

### Study Identification and Characteristics

**Fig. 1:**
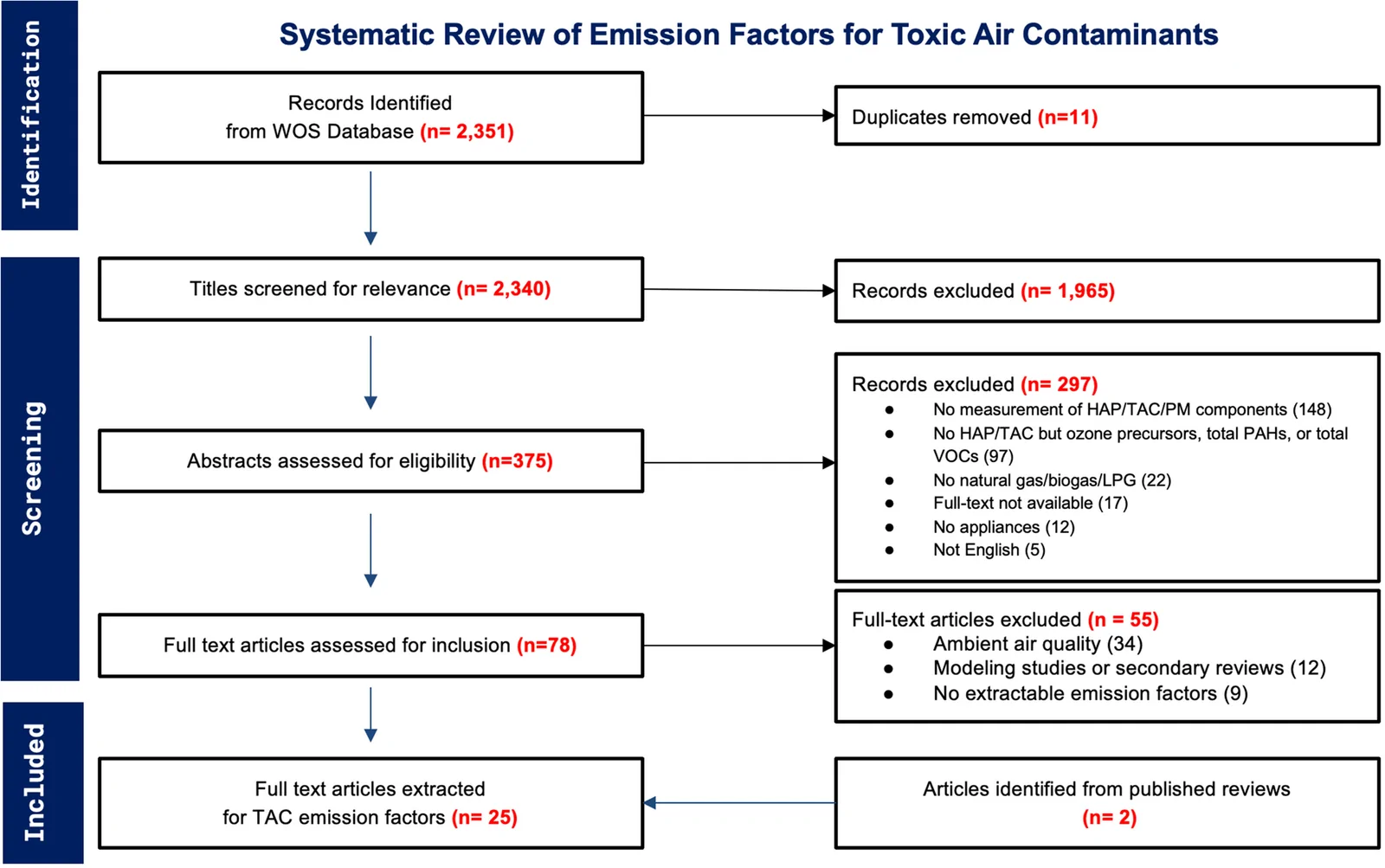
Study identification and selection process for the systematic review of TACs emission factors. The figure presents the study selection process for this systematic review of TAC emission factors from gas appliances. The flow diagram is organized into three sequential phases: Identification, Screening, and Included

The Web of Science search returned 2,351 records. After removing 11 duplicates, 2,340 titles were screened and 1,965 were excluded as clearly outside scope. Seventy-eight full-text articles were then assessed for eligibility. Of these, 55 were excluded: 34 reported ambient air quality measurements without appliance-level source apportionment, 12 were modeling studies or secondary reviews without original emission measurements, and 9 lacked extractable EF data in an appropriate format. Twenty-two additional studies identified through reference-list screening were assessed, of which two met all criteria. A total of 25 studies met all inclusion criteria and contributed TAC EF data to the synthesis (Fig. 1).

**Fig. 2:**
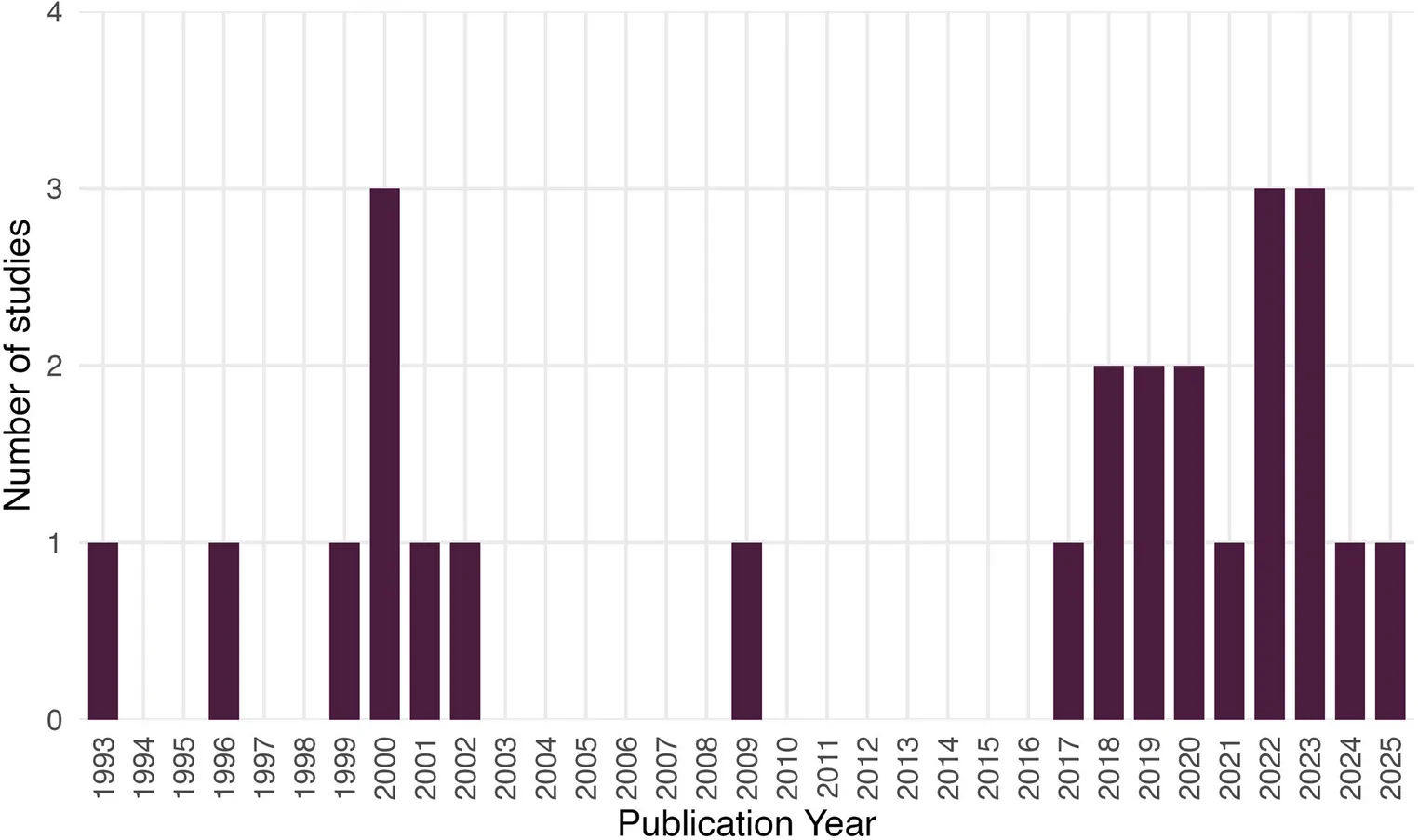
Description of study characteristics for the included studies (*N* = 25). This figure summarizes the characteristics of studies included in this review, expressed as the percentage of studies falling into each category. Panels **A**, **B**, **C**, and **D **respectively show the distribution of study geographic locations, study setting, fuel type, and appliance type. Individual studies may contribute to multiple categories where applicable

**Fig. 3:**
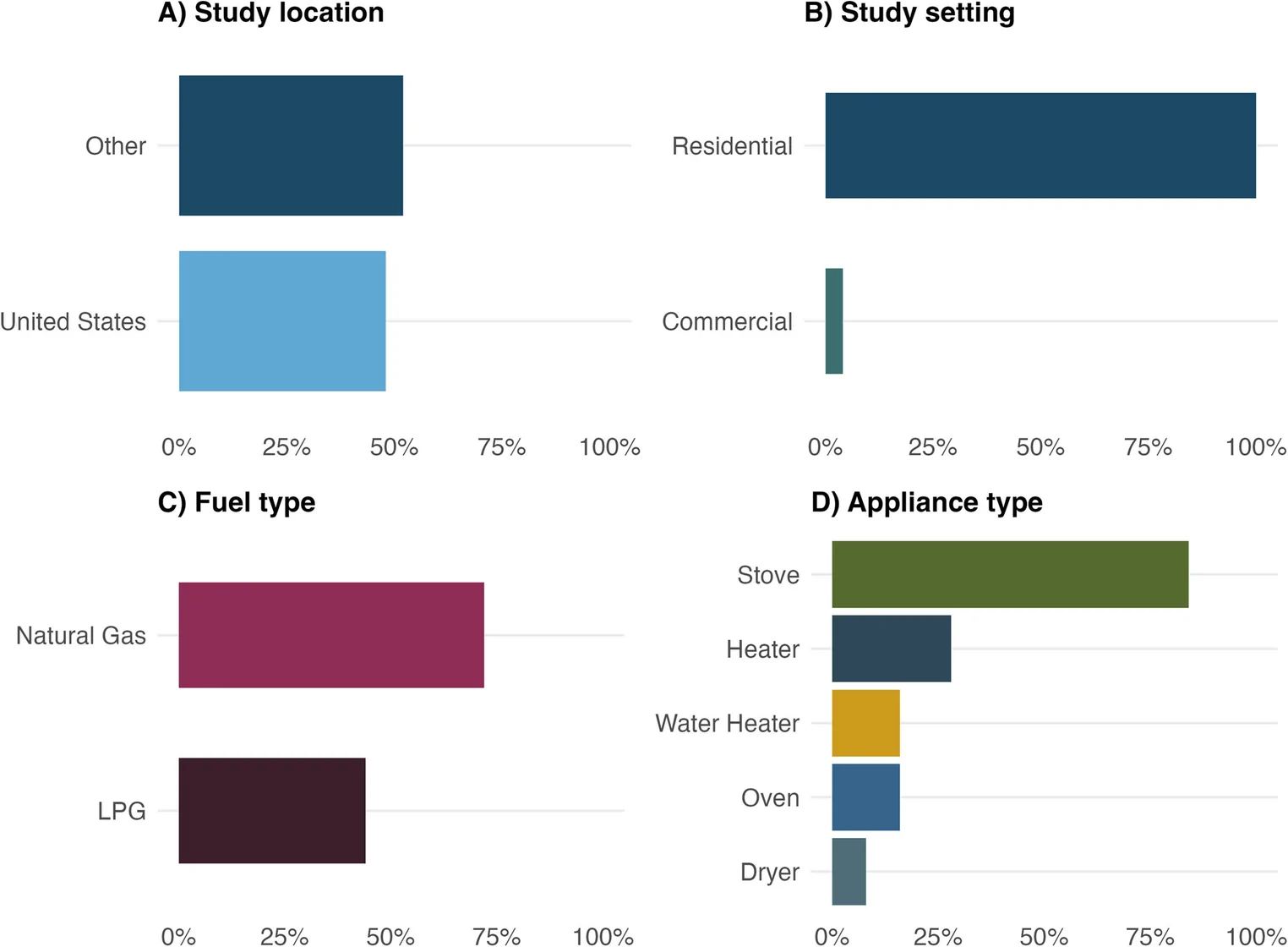
Publication timeline for studies included in this review (*N* = 25). This figure shows the number of studies published per year that met the inclusion criteria for this systematic review of TAC emission factors from gas appliances. The primary literature search spanned 2000–2024; studies appearing on the timeline prior to 2000 were identified through reference lists and prior review articles rather than the systematic search

The geographic distribution of studies was heterogeneous (Fig. 2A); approximately half were conducted outside the United States and California accounted for a slightly larger share of U.S.-based studies than any other state. All studies included measurements conducted in residential settings, with one study additionally evaluating commercial appliances (Fig. 2B). Natural gas was the most commonly investigated fuel type (Fig. 2C). Studies examining LPG were less common and few studies directly compared fuels within the same experimental design. The literature was heavily concentrated on gas stoves, which were evaluated in over 75% of studies (Fig. 2D). Other appliances, including heaters, water heaters, ovens, and dryers, were less frequently examined. Research on TAC emission factors from gas appliances has increased markedly in recent years (Fig. 3).

After sporadic publications in the 1990s and early 2000s, relatively few studies appeared until around 2017, after which annual publication counts rose steadily. Most studies in the review were published between 2018 and 2023, reflecting growing scientific and policy interest in indoor emissions from gas appliances.

### Evidence of Non-Zero TAC Emissions

Evidence of nonzero TAC emissions was identified for at least 41 compounds across leakage and combustion pathways (Table 1). BTEX compounds showed evidence across both fuel types and pathways, reflecting their presence in unburned gas and their formation during high-temperature combustion. Formaldehyde, acetaldehyde, and acrolein were documented from combustion of both natural gas and LPG appliances. LPG combustion studies documented a broader set of compounds than natural gas studies, including PAHs and halogenated compounds not measured in most natural gas-focused investigations. Absence of documented evidence for a given compound-pathway combination should not be interpreted as absence of emissions.

**Table 1:** Evidence of non-zero TAC emissions by fuel type and pathway, independent of appliance type. Two fuel categories (natural gas and propane/LPG) are presented, each subdivided into two pathways: leakage (unburned fuel emissions) and combustion and formation (pollutants generated during appliance operation). For every pollutant–fuel–pathway combination, cells indicate whether at least one study has reported evidence of non-zero emissions

Fuel Type	Evidence of nonzero emissions?^1^			
	Natural gas		Propane/LPG	
Pathway	Leakage	Combustion & formation	Leakage	Combustion & formation
Benzene	Yes^2^	Yes	No	Yes
Toluene	Yes^2^	Yes	No	Yes
Ethylbenzene	Yes^2^	Yes	No	Yes
Xylenes	Yes^2^	Yes	No	Yes
Hexane	Yes^2^	No	No	No
Acenaphthene	No	No	No	Yes
Acenaphthylene	No	Yes	No	Yes
Acetaldehyde	No	Yes	No	Yes
Acrolein	No	No	No	Yes
Anthracene	No	No	No	Yes
Benz[a]anthracene	No	No	No	Yes
Benzo[a]pyrene	No	No	No	Yes
Benzo[a, h]anthracene	No	No	No	Yes
Benzo[b]fluoranthene	No	No	No	Yes
Benzo[e]pyrene	No	No	No	Yes
Benzo[b]fluoranthene	No	No	No	Yes
Benzo[ghi]fluoranthene	No	No	No	Yes
Benzo[ghi]perylene	No	No	No	Yes
Benzo[k]fluoranthene	No	No	No	Yes
Chrysene	No	No	No	Yes
Coronene	No	No	No	Yes
Crotonaldehyde	No	Yes	No	Yes
Cyclohexane	Yes^2^	No	No	Yes
Dibenzo[a, h]anthrancene	No	No	No	Yes
Fluoranthene	No	No	No	Yes
Fluorene	No	No	No	Yes
Formaldehyde	No	Yes	No	Yes
Indeno [1,2,3-cd] pyrene	No	No	No	Yes
Naphthalene	No	Yes	No	Yes
1-Methylchrysene	No	No	No	Yes
1-Methylnaphthalene	No	Yes	No	Yes
2,6-Dimethylnaphthalene	No	No	No	Yes
2-Methylnaphthalene	No	Yes	No	Yes
2-butanone	No	No	No	Yes
Methylfluorene	No	No	No	Yes
Perylene	No	No	No	Yes
Phenanthrene	No	No	No	Yes
Polychlorinated Dibenzo-p-Dioxins and Polychlorinated Dibenzofurans (PCDD/PCDF)	No	Yes	No	No
Propionaldehyde	No	Yes	No	Yes
Propylene	No	Yes	No	No
Pyrene	No	No	No	Yes
Retene	No	No	No	Yes
Carbon tetrachloride	No	Yes^3^	No	No
Chloroethane	No	Yes^3^	No	No
Chloromethane	No	Yes^3^	No	No
1,1,2,2-Tetrachloroethane	No	Yes^3^	No	No
Methane^4^	Yes	Yes	No	Yes

1. Evidence of nonzero emissions entails at least one peer reviewed scientific study that met our inclusion criteria. Absence of evidence does not mean absence of emissions.
2. Inferred from methane leakage and composition of unburned natural gas by Lebel et al.
3. Evidence is from a single study that grouped washer & dryer emissions together and did not differentiate between emissions from appliance operation vs. combustion.
4. Methane is not a TAC but is included here as a proxy for leakage.

Only formaldehyde (*n* = 14 EF measurements) and benzene (*n* = 10) had sufficient data to support quantitative characterization of EF variability across studies and fuel-appliance combinations (Table 2). All other identified TACs had three or fewer measurements, and the majority had only one or two. This data sparsity has direct regulatory consequences: for most of the 41 documented TAC emissions, the evidence base cannot support calculation of a reliable central-tendency EF, and standard-setting based on available data for these compounds would carry high uncertainty. 

**Table 2:** Summary statistics of standardized emission factors (EFs) for TACs with sufficient EF data. This table presents descriptive statistics of TACs with at least three available EF measurements, enabling calculation of variability metrics such as the standard deviation. For each TAC, the number of EF measurements (n), mean, standard deviation (SD), median, interquartile range (IQR; the range between the 25th and 75th percentiles, reflecting the spread of the central 50% values), and minimum and maximum values are reported. All EFs are expressed in standardized units (mg/MJ of fuel burned). TACs with fewer than three EFs were excluded from this table due to insufficient data to characterize variability

TAC	*n*	Mean	SD	Median	IQR	Min	Max
Formaldehyde	14	1.40	1.10	1.28	1.90	0.073	3.24
Benzene	10	0.0249	0.0293	0.0107	0.0338	0.00173	0.0921

### Formaldehyde Emission Factors

**Fig. 4:**
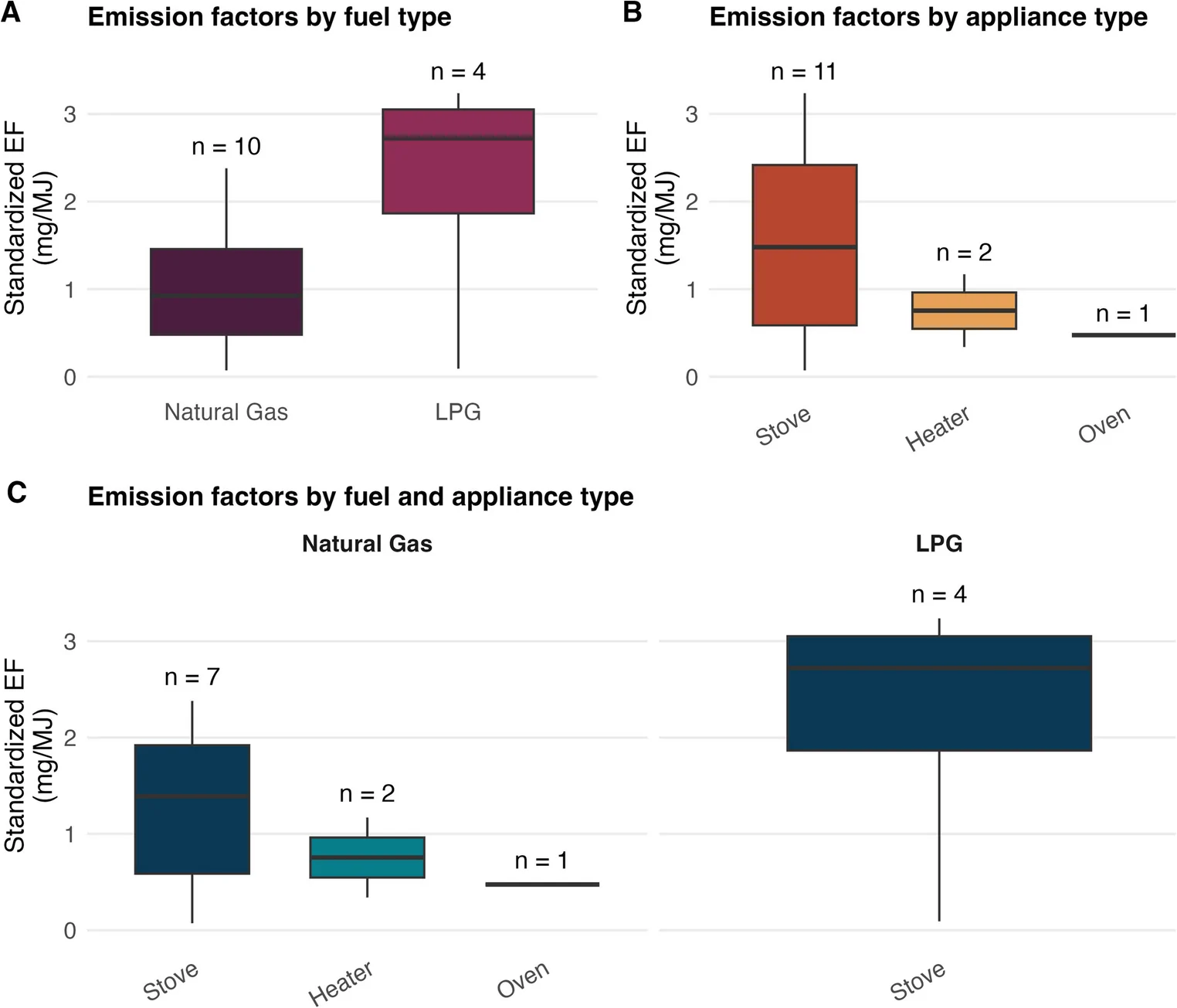
Distribution of formaldehyde emission factors (EFs) by fuel type and appliance category. Boxplots of standardized formaldehyde EFs (mg/MJ), respectively stratified by (**A**) fuel type (liquefied petroleum gas [LPG] and natural gas), (**B**) appliance type (stoves and heaters), and (**C**) joint fuel type and appliance category. Boxes represent the interquartile range (IQR), center lines indicate the median, and whiskers extend to the range of observed values. Sample sizes (n), representing the number of EF measurements, are shown above each category

Formaldehyde was the best-characterized TAC in the reviewed literature, with 14 EF measurements identified across 6 studies and across multiple fuel types and appliance categories (Fig. 4). Across all available measurements, EFs ranged from 0.073 to 3.24 mg/MJ, with a mean of 1.40 mg/MJ (standard deviation [SD] = 1.10), a median of 1.28 mg/MJ, and an interquartile range of 1.90 mg/MJ. The range spans nearly two orders of magnitude, underscoring the high variability intrinsic to appliance emission measurements and the risk of placing regulatory reliance on any single representative value.

Four formaldehyde EF measurements were identified for LPG appliances, all derived from stove combustion experiments, ranging from 0.193 to 3.24 mg/MJ. [27–29] Ten measurements were identified for natural gas appliances across multiple studies and a broader range of appliance types, [25, 28–31] with EFs ranging from 0.073 to 2.38 mg/MJ. LPG measurements tended toward higher values but the distributions overlapped substantially, and the limited sample size prevents definitive conclusions about systematic fuel-type differences in formaldehyde emission rates. By appliance type, eleven measurements corresponded to stoves, spanning the full observed range. Two natural gas space heater measurements (0.34 and 1.17 mg/MJ30) and one natural gas oven measurement (0.475 mg/MJ25) fell within the stove distribution, suggesting broadly comparable formaldehyde emission potential across appliance types, though the evidence base for non-stove appliances is insufficient to support quantitative conclusions. No formaldehyde EF data were identified for heaters, water heaters, or commercial appliances of any category.

This variability is plausible: formaldehyde yields are sensitive to combustion temperature, air-to-fuel ratio, burner design, and appliance maintenance, all of which vary across the experimental conditions represented in the literature. Singer et al. (2009) documented within-appliance EF variation of 0.09 to 4.67 mg/MJ across operating conditions for a single stove. This range exceeds the entire cross-study distribution and has implications for health risk assessment and standard design given that formaldehyde is a confirmed human carcinogen.

### Benzene Emission Factors

**Fig. 5:**
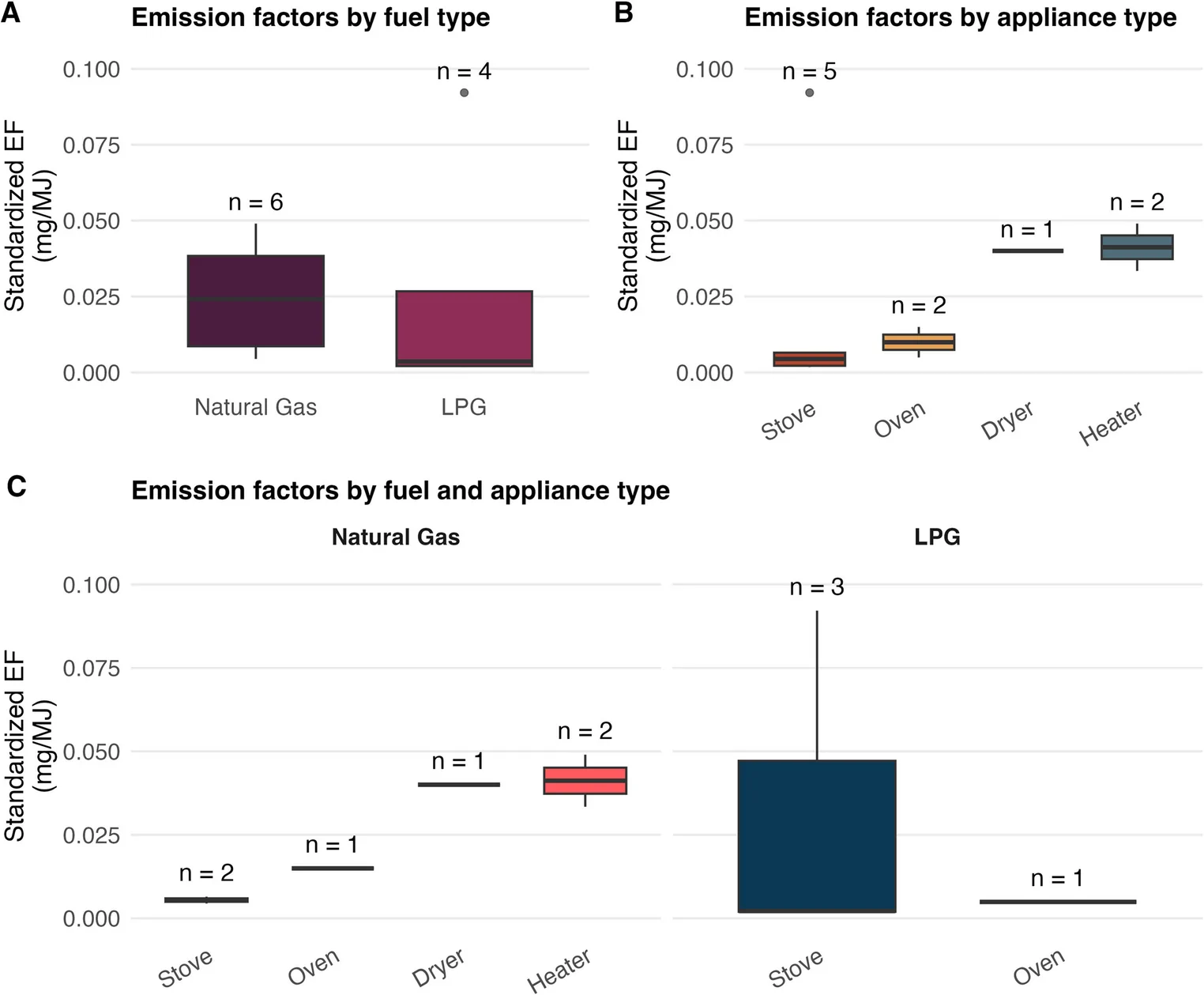
Distribution of benzene emission factors (EFs) by fuel type and appliance category. Boxplots of standardized benzene EFs (mg/MJ), respectively stratified by (**A**) fuel type (liquefied petroleum gas [LPG]/propane and natural gas), (**B**) appliance type (stoves and dryers), and (**C**) joint fuel type and appliance category. Boxes represent the interquartile range (IQR), center lines indicate the median, and whiskers extend to the range of observed values excluding outliers. Sample sizes (n), representing the number of EF measurements, are shown above each category

Ten benzene EF measurements (originating from 4 studies) were identified across two fuel types and multiple appliance categories (Fig. 5). Across all measurements, benzene EFs ranged from 0.00173 to 0.0921 mg/MJ (mean 0.0249 mg/MJ, SD = 0.0293, median 0.0107 mg/MJ, IQR = 0.0338 mg/MJ). The distribution was markedly right-skewed: most measurements clustered at relatively low values below 0.05 mg/MJ, but a single LPG stove measurement extended the upper tail substantially, and the distribution mean was well above the median as a result. Four EF measurements were from LPG appliances and six from natural gas appliances, with overlapping distributions at lower values but a broader LPG range. [7, 27, 32, 33].

By appliance type, five EFs corresponded to stoves, two to ovens, one to a natural gas dryer, and two to natural gas heater (including a commercial heater). The dryer measurement (0.040 mg/MJ32) was among the higher observed values. This is notable because dryers represent one of the least-studied appliance categories and often operate without kitchen exhaust ventilation. The heater measurements (0.0334 and 0.0490 mg/MJ) were also among the higher natural gas EFs observed, with the higher value measured from a commercial heating appliance. [33 ] The two oven measurements (LPG: 0.00489 mg/MJ; natural gas: 0.01494 mg/MJ7) fell within the stove distribution. As an IARC Group 1 carcinogen with no safe exposure threshold, even the lower benzene EFs could translate to non-trivial incremental cancer risk under chronic residential exposure.

### PAHs and Other TACs

PAHs were documented across four studies and two fuel types, although the evidence was dominated by LPG-fueled stoves. [27, 33–35] Lower molecular weight PAHs, such as naphthalene, methylnaphthalenes, fluorene, acenaphthene, and acenaphthylene, exhibited EFs in the range of approximately 0.000046 to 0.048 mg/MJ, with the limited measurements from natural gas heaters falling at the lower end of this range (0.000074–0.000209 mg/MJ). Intermediate molecular weight species including phenanthrene, anthracene, fluoranthene, and pyrene ranged from approximately 0.000187 to 0.031 mg/MJ, while higher molecular weight PAHs, including benz[a]anthracene, chrysene, benzo[a]pyrene, indeno[1,2,3-cd]pyrene, and dibenzo[a, h]anthracene, several of which have been classified as known, probable, or possible human carcinogens, [36] were generally reported at lower EF levels, typically below 0.005 mg/MJ. Overall, PAH characterization remains heavily concentrated on LPG stoves, with limited data for natural gas and non-cooking appliances. Other TACs documented in this review include acetaldehyde (LPG stoves: 0.64–3.06 mg/MJ [27, 29]), acrolein, propionaldehyde, and crotonaldehyde from both LPG and natural gas stoves, as well as aromatic VOCs including toluene, ethylbenzene, and xylenes across fuel and appliance types. [27, 32] Toluene EFs ranged from 0.044 mg/MJ for an LPG stove to 0.098 mg/MJ for a residential natural gas heater, while the commercial heater measurement was below the detection limit. [27, 33] Natural gas dryers emitted halogenated compounds including chloromethane, chloroethane, and carbon tetrachloride, compounds not measured in most stove-focused studies and whose presence underscores the need for systematic non-cooking appliance characterization. Compound-level EF data for PAHs and other TACs are provided in full in Supplementary Sections S1 and S2.

### Inferring TAC Emissions from Natural Gas Leakage and Incomplete Combustion

Given the limited direct measurement data available for many appliance categories and pollutants, two inference pathways offer practical tools for partially filling evidence gaps and extending the regulatory applicability of the existing dataset. Both approaches leverage widely measured proxy variables to estimate TAC emission rates in settings where direct TAC characterization is absent or infeasible.

Because consumer-grade natural gas is 90%+ methane, the first pathway uses methane as a tracer to scale accompanying TACs also present in consumer-grade natural gas. [23, 37] To infer TAC emissions from a natural gas sourced-leak that is primarily methane (> 90%), a molar ratio of a TAC measured in unburned natural gas to methane measured in unburned natural gas can be assumed to be conserved in an open path leak at typical operating pressures of residential gas systems (e.g., 20 mbar gauge pressure). Therefore, where appliance methane leakage rates are known (which are much more readily available), TAC emission rates can be inferred. For example, measurements by Lebel et al. (2022b) showed benzene concentrations in whole, unburned natural gas throughout California pipeline end use systems (i.e., samples collected from gas stoves) ranging from 0.7 to 12 ppmv (max: 66 ppmv). Applying previously published behind-the-meter methane leakage estimates, Lebel et al. (2022b), estimated that 310 (95% CI: 140 − 980) kg benzene yr–1 is emitted in California from whole-house residential leaks, with an estimated 63 (95% CI: 32 − 150) kg benzene per year emitted from gas stove steady-state-off leakage alone. Lebel et al. (2022) identified seven TACs in California pipeline gas at concentrations sufficient to generate exposure-relevant leakage-derived emission rates. Similar patterns of toxic air contaminant occurrence and exposure-relevant gas leakage have been observed elsewhere in North America. [37] The full derivation and example calculations are in Supplementary Section S1.

The second pathway uses CO as a surrogate for incomplete combustion. CO is routinely measured in appliance emission studies and is an established indicator of combustion completeness. Kashtan et al. (2023) analyzed 80 natural gas and propane burners and ovens throughout California and reported a statistically significant power-law relationship between benzene and CO emission rates (R² = 0.57), demonstrating that CO measurements can serve as a practical surrogate for benzene emission estimation in field settings where direct TAC sampling is impractical. This relationship holds important potential for extending the regulatory utility of existing CO monitoring datasets to estimate benzene burdens at the population level. However, the specific functional relationship requires validation across a broader range of appliance types, fuel compositions, and combustion conditions before being applied broadly in regulatory frameworks. The full inference equation derivation and an illustrative worked example are provided in Supplementary Section S2.

Taken together, both inference pathways provide order-of-magnitude estimates appropriate for inventory development and screening-level exposure assessment. They are not substitutes for direct EF measurement but represent a practical toolset for regulators and researchers operating in data-sparse environments. Their further development and systematic validation should be a near-term priority for agencies developing TAC emission inventories for the residential sector.

## Discussion

### Policy and Regulatory Implications

#### Confirming the Regulatory Basis for TAC Standards

The evidence synthesized here is sufficient to establish that formaldehyde and benzene, both confirmed human carcinogens, are routinely emitted by residential gas appliances at concentrations that could translate to non-trivial health risk increments under chronic exposure. The same applies to acetaldehyde, an IARC Group 1 carcinogen detected at some of the highest EF values in this review, and to PAHs and other probable carcinogens with documented combustion-pathway emissions. CARB and other state and federal agencies have evidence for including TAC performance criteria in next-generation appliance efficiency and emissions standards. This review provides the most comprehensive synthesis to date of measured TAC EFs from gas appliances across fuel and appliance types, demonstrating that the absence of prior systematic synthesis does not reflect an absence of evidence, but rather a gap that this work addresses. 

#### Accounting for the Appliance-Type Evidence Asymmetry

The overwhelming focus on cooking stoves in the TAC EF literature creates a systematic risk of underestimating whole-home TAC burdens from gas use. Heaters and space heaters are the primary combustion source in the majority of gas-heated homes, operating for thousands of hours per heating season in occupied living spaces that may have limited mechanical ventilation. Water heaters installed in attached utility closets or garages can contribute combustion-related emissions to occupied spaces through building envelope pressure dynamics and combustion air pathways. Clothes dryers, while typically vented to the outdoors, can also expose occupants through backdrafting events or in installations with non-compliant venting. Although the limited available measurements from heaters demonstrate emissions of benzene, PAHs, and other TACs, the evidence for non-cooking appliances remains very limited compared with stoves. Additional measurement campaigns for these underrepresented categories are necessary before appliance-specific standards for those sources are developed. 

#### Designing Standards that Reflect Real-World Emission Variability

EFs for well-characterized TACs vary by one to two orders of magnitude across appliances and operating conditions. Emission standards built around central-tendency EFs will fail to protect against high-emitting units that may dominate population exposure in the tail of the distribution. Vehicle emissions regulation addresses similar variability through certification test designs that must be met at the 95th percentile of the emission distribution and through in-use compliance surveillance programs. Similar tail-emission thresholds, lot-acceptance testing requirements, and in-use compliance audits could be adapted for appliance TAC standards, but would require measurement campaigns specifically designed to characterize the full emission distribution rather than mean values alone. 

#### Strengthening TAC Inventories Through Inference and Monitoring

The EPA’s National Emissions Inventory currently lacks reliable TAC estimates for the residential gas combustion sector, relying on dated or absent EF data for many compounds. [38] Embedding validated CO-TAC and methane-leakage inference relationships into NEI methodology development would substantially improve inventory accuracy and enable more robust cumulative impact and environmental justice analyses. In California, existing methane leak detection and reporting programs implemented by utilities present an opportunity to estimate leakage-related TAC emissions through the integration of methane measurements and gas composition profiles.

### Uncertainties and Research Priorities

EF estimates reported in this review carry substantial uncertainty that users must understand before applying them in exposure assessment or standard-setting contexts. The most fundamental limitation is data sparsity: for the vast majority of the 41 documented TAC emissions, EFs derive from only one or two independent studies and in many cases from a single experimental trial under specific controlled conditions. The scientific standard for robust EF characterization (i.e., multiple measurements across diverse appliance samples, fuel compositions, and operating conditions) is met only by formaldehyde and benzene in this dataset, and even for these compounds the evidence base is thin compared to better-studied emission source categories such as on-road mobile sources or large industrial point sources.

Methodological heterogeneity across studies compounds this uncertainty. Included studies varied substantially in sampling instrumentation and detection limits, combustion test protocols (simulated cooking, steady-state burner tests, cyclic operating conditions), measurement duration and averaging periods, ventilation conditions, and criteria for appliance selection. These differences make it difficult to distinguish genuine emission differences from methodological differences, and mean that direct numerical comparison of EFs across studies requires caution even after unit harmonization. The statistical distribution of appliance EFs adds another regulatory-relevant complication: appliance emission distributions are typically right-skewed and approximately lognormal, such that arithmetic means overstate typical emissions while masking the significance of high-emitting units in the tail of the distribution that may disproportionately contribute to population exposure.

Given this uncertainty, four targeted research investments represent the highest-priority opportunities to strengthen the evidence base. First, multi-appliance field measurement campaigns should prioritize non-cooking appliances, particularly heaters, water heaters, and dryers, which remain sparsely characterized relative to cooking appliances despite their contribution to whole-home gas combustion. Second, replication of existing key EF estimates across multiple laboratories and appliance sets is essential to establish representativeness and reduce reliance on single-source values. Third, future measurement studies should systematically co-measure TACs alongside CO and methane under matched conditions, enabling robust validation and extension of inference relationships. Fourth, integrating laboratory EF measurements with paired in-home field measurements in real residential settings, with explicit sampling in environmentally overburdened communities, would substantially improve both the external validity of EF data and the equity dimensions of TAC burden assessment.

## Conclusion

This systematic review provides the most comprehensive synthesis to date of toxic air contaminant (TAC) emission factors from residential and commercial fossil fuel gas appliances. Across 25 studies reporting TACs from appliance emissions, the evidence shows that TACs released through leakage and combustion are quantifiable and likely significant public health concerns. The findings support regulatory attention to both combustion- and leakage-related emissions, highlight the need for standardized emission factor methodologies, and identify formaldehyde and benzene as priority pollutants for future measurement and mitigation efforts. Given the potential cumulative burden of indoor air pollution, particularly in disadvantaged communities, expanded monitoring, appliance testing, and systematic inventory development are needed to better characterize emissions and inform effective public health and policy interventions. 

## Key References

- Kashtan YS, Nicholson M, Finnegan C, Ouyang Z, Lebel ED, Michanowicz DR et al. Gas and Propane Combustion from Stoves Emits Benzene and Increases Indoor Air Pollution. Environ Sci Technol 2023; 57: 9653–9663.
  - ○ A landmark empirical study measuring benzene and other toxic emissions from natural gas and propane burners and ovens across California, demonstrating that combustion of both fuels generates benzene emissions indoors, and establishing a relationship between benzene and CO emission rates that provides a practical surrogate-based inference method for settings where direct TAC sampling is impractical.
- Lebel E, Michanowicz D, Bilsback K, Hill L, Goldman J, Domen J et al. Composition, Emissions, and Air Quality Impacts of Hazardous Air Pollutants in Unburned Natural Gas from Residential Stoves in California. Environ Sci Technol 2022; 56.
  - ○ Characterizes the BTEX and other hazardous air pollutant content of unburned natural gas sampled at California residential stoves, establishing that routine appliance leakage in the off state releases meaningful quantities of benzene and identifying leakage as an underappreciated route of TAC exposure independently of combustion.
- Bilsback K, Dahlke J, Fedak K, Good N, Hecobian A, Herckes P et al. A Laboratory Assessment of 120 Air Pollutant Emissions from Biomass and Fossil Fuel Cookstoves. Environ Sci Technol 2019; 53: 7114–7125.
  - ○ Provides one of the most comprehensive multi-pollutant laboratory emission factor datasets in this review, reporting emission factors for a wide array of TACs including carbonyls, aromatic hydrocarbons, and PAHs from LPG and biomass stoves under controlled conditions, making it a primary source for PAH and other TAC data synthesized throughout this paper.
•Singer BC, Apte M, Black D, Hotchi T, Lucas D, Lunden M et al. Natural Gas Variability in California: Environmental Impacts and Device Performance: Experimental Evaluation of Pollutant Emissions from Residential Appliances. Lawrence Berkeley National Laboratory, 2009.
  - ○ An early and foundational laboratory study documenting emission factors for formaldehyde and other pollutants from natural gas residential appliances, notable for demonstrating extreme within-appliance variability across operating conditions on a single stove, with important implications for how appliance emission standards must account for real-world variability.
•Rowland ST, Lebel ED, Goldman JSW, Domen JK, Bilsback KR, Ruiz A et al. Downstream natural gas composition across U.S. and Canada: implications for indoor methane leaks and hazardous air pollutant exposures. Environ Res Lett 2024; 19: 064064.
  - ○ Extends California-focused gas composition findings to a broader North American context, confirming that exposure-relevant hazardous air pollutant concentrations in unburned pipeline gas and the associated leakage-pathway TAC risks are not California-specific.

## Supplementary Information

Below is the link to the electronic supplementary material.

Supplementary Material 1

Supplementary Material

## Data Availability

No datasets were generated or analysed during the current study.

## References

[1] US EIA. Residential Energy Consumption Survey (RECS) – 2020 RECS Survey Data. 2020. https://www.eia.gov/consumption/residential/data/2020/index.php?view=state%5C (accessed 4 Mar2026).

[2] US EIA. Natural gas explained - Use of natural gas. 2024. https://www.eia.gov/energyexplained/natural-gas/use-of-natural-gas.php Accessed 4 Mar 2026.

[3] US EIA. Number of Natural Gas Consumers. 2026. https://www.eia.gov/dnav/ng/ng_cons_num_a_EPG0_VN3_Count_a.htm Accessed 16 Feb 2026.

[4] Brandt AR, Heath GA, Kort EA, O’Sullivan F, Pétron G, Jordaan SM, et al. Methane Leaks from North American Natural Gas Systems. Science. 2014;343:733–5. 24531957 10.1126/science.1247045

[5] PHMSA. Annual Report Mileage for Gas Distribution Systems. 2026. https://www.phmsa.dot.gov/data-and-statistics/pipeline/annual-report-mileage-gas-distribution-systems Accessed 4 Mar 2026.

[6] Fischer ML, Chan WR, Delp W, Jeong S, Rapp V, Zhu Z. An Estimate of Natural Gas Methane Emissions from California Homes. Environ Sci Technol. 2018;52:10205–13. 30071722 10.1021/acs.est.8b03217

[7] Kashtan YS, Nicholson M, Finnegan C, Ouyang Z, Lebel ED, Michanowicz DR, et al. Gas and Propane Combustion from Stoves Emits Benzene and Increases Indoor Air Pollution. Environ Sci Technol. 2023;57:9653–63. 37319002 10.1021/acs.est.2c09289 PMC10324305

[8] Propper R, Wong P, Bui S, Austin J, Vance W, Alvarado A, et al. Ambient and emission trends of toxic air contaminants in California. Environ Sci Technol. 2015;49:11329–39. 26340590 10.1021/acs.est.5b02766

[9] Lin W, Brunekreef B, Gehring U. Meta-analysis of the effects of indoor nitrogen dioxide and gas cooking on asthma and wheeze in children. Int J Epidemiol. 2013;42:1724–37. 23962958 10.1093/ije/dyt150

[10] Chemicals IARC., Industrial Processes and Industries Associated with Cancer in Humans (IARC Monographs Volumes 1 to 29). https://publications.iarc.who.int/Book-And-Report-Series/Iarc-Monographs-Supplements/Chemicals-Industrial-Processes-And-Industries-Associated-With-Cancer-In-Humans-IARC-Monographs-Volumes-1-To-29--1982 Accessed 2 June 2026.

[11] Feng Y, Li Z, Li W. Polycyclic Aromatic Hydrocarbons (PAHs): Environmental Persistence and Human Health Risks. Nat Prod Commun. 2025;20:1934578X241311451.

[12] Marques MM, Beland FA, Lachenmeier DW, Phillips DH, Chung F-L, Dorman DC, et al. Carcinogenicity of acrolein, crotonaldehyde, and arecoline. Lancet Oncol. 2021;22:19–20. 33248467 10.1016/S1470-2045(20)30727-0

[13] Adamkiewicz G, Spengler JD, Harley AE, Stoddard A, Yang M, Alvarez-Reeves M, et al. Environmental Conditions in Low-Income Urban Housing: Clustering and Associations With Self-Reported Health. Am J Public Health. 2014;104:1650–6. 24028244 10.2105/AJPH.2013.301253 PMC3954449

[14] Benfer EA, Gold AE. There’s No Place Like Home: Reshaping Community Interventions and Policies to Eliminate Environmental Hazards and Improve Population Health for Low-Income and Minority Communities. Rochester, NY: Social Science Research Network; 2017. https://papers.ssrn.com/abstract=3006099. accessed 22 Apr2021.

[15] Grineski SE, Hernández AA. Landlords, fear, and children’s respiratory health: an untold story of environmental injustice in the central city. Local Environ. 2010;15:199–216.

[16] Krieger J, Higgins DL. Housing and Health: Time Again for Public Health Action. Am J Public Health. 2002;92:758–68. 11988443 10.2105/ajph.92.5.758 PMC1447157

[17] Acquaye L. Low-income homeowners and the challenges of home maintenance. Community Dev. 2011;42:16–33.

[18] Lane K, Daouda M, Yuan A, Olson C, Smalls-Mantey L, Siegel E, et al. Readiness for a clean energy future: Prevalence, perceptions, and barriers to adoption of electric stoves and solar panels in New York city. Energy Policy. 2024;194:114301. 39463762 10.1016/j.enpol.2024.114301 PMC11507541

[19] Kashtan Y, Nicholson M, Finnegan CJ, Ouyang Z, Garg A, Lebel ED, et al. Nitrogen dioxide exposure, health outcomes, and associated demographic disparities due to gas and propane combustion by U.S. stoves. Sci Adv. 2024;10:eadm8680. 38701214 10.1126/sciadv.adm8680 PMC11068006

[20] CARB Identified Toxic Air Contaminants | California Air Resources Board. https://ww2.arb.ca.gov/resources/documents/carb-identified-toxic-air-contaminants Accessed 10 June 2026.

[21] Francoeur CB, McDonald BC, Gilman JB, Zarzana KJ, Dix B, Brown SS, et al. Quantifying Methane and Ozone Precursor Emissions from Oil and Gas Production Regions across the Contiguous US. Environ Sci Technol. 2021;55:9129–39. 34161066 10.1021/acs.est.0c07352

[22] Lebel E, Finnegan C, Ouyang Z, Jackson R. Methane and NOx Emissions from Natural Gas Stoves, Cooktops, and Ovens in Residential Homes. Environ Sci Technol. 2022;56:2529–39. 35081712 10.1021/acs.est.1c04707

[23] Lebel E, Michanowicz D, Bilsback K, Hill L, Goldman J, Domen J, et al. Composition, Emissions, and Air Quality Impacts of Hazardous Air Pollutants in Unburned Natural Gas from Residential Stoves in California. Environ Sci Technol. 2022;56. 10.1021/acs.est.2c02581. PMC9671046 36263944

[24] Michanowicz DR, Dayalu A, Nordgaard CL, Buonocore JJ, Fairchild MW, Ackley R, et al. Home is where the pipeline ends: characterization of volatile organic compounds present in natural gas at the point of the residential end user. Environ Sci Technol. 2022;56:10258–68. 35762409 10.1021/acs.est.1c08298 PMC9301916

[25] Singer BC, Apte M, Black D, Hotchi T, Lucas D, Lunden M, et al. Natural Gas Variability in California: Environmental Impacts and Device Performance: Experimental Evaluation of Pollutant Emissions from Residential Appliances. Lawrence Berkeley Natl Lab. 2009. 10.2172/980736.

[26] US EPA. Source Classification Codes (SCCs). 2016. https://sor-scc-api.epa.gov/sccwebservices/sccsearch/ Accessed 4 Mar 2026.

[27] Bilsback K, Dahlke J, Fedak K, Good N, Hecobian A, Herckes P, et al. A Laboratory Assessment of 120 Air Pollutant Emissions from Biomass and Fossil Fuel Cookstoves. Environ Sci Technol. 2019;53:7114–25. 31132247 10.1021/acs.est.8b07019

[28] Ye J, Shi S, Yu T, Liu C, Li J, Ding Z, et al. Emissions of formaldehyde and nitrogen dioxide from liquefied petroleum gas combustion. Process Saf Environ Prot. 2023;177:1225–33.

[29] Zhang J, Smith KR. Emissions of carbonyl compounds from various cookstoves in China. Environ Sci Technol. 1999;33:2311–20.

[30] Traynor GW, Apte MG, Chang G-M. Pollutant Emission Factors from Residential Natural Gas Appliances: A Literature Review. Lawrence Berkeley National Laboratory. 1996.

[31] Zheng Z, Zhang H, Qian H, Li J, Yu T, Liu C. Emission characteristics of formaldehyde from natural gas combustion and effects of hood exhaust in Chinese kitchens. Sci Total Environ. 2022;838:156614. 35691355 10.1016/j.scitotenv.2022.156614

[32] Van Winkle M, Scheff P. Volatile Organic Compounds, Polycyclic Aromatic Hydrocarbons and Elements in the Air of Ten Urban Homes. Indoor Air - Int J Indoor Environ Health. 2001;11:49–64. 10.1034/j.1600-0668.2001.011001049.x 11235231

[33] Lindberg J, Trojanowski R, Galvin S, Butcher T. Climate and health-relevant pollutant emissions from oil and natural gas boilers. J Air Waste Manag Assoc. 2025;75:789–807. 40910746 10.1080/10962247.2025.2547634

[34] Champion W, Hays M, Williams C, Virtaranta L, Barnes M, Preston W, et al. Cookstove Emissions and Performance Evaluation Using a New ISO Protocol and Comparison of Results with Previous Test Protocols. Environ Sci Technol. 2021;55:15333–42. 34714622 10.1021/acs.est.1c03390 PMC8855438

[35] Shen G, Preston W, Ebersviller SM, Williams C, Faircloth JW, Jetter JJ, et al. Polycyclic aromatic hydrocarbons in fine particulate matter emitted from burning kerosene, liquid petroleum gas, and wood fuels in household cookstoves. Energy Fuels. 2017;31:3081–90. 30245546 10.1021/acs.energyfuels.6b02641 PMC6145494

[36] Straif K, Baan R, Grosse Y, Secretan B, Ghissassi FE, Cogliano V. Carcinogenicity of polycyclic aromatic hydrocarbons. Lancet Oncol. 2005;6:931–2. 16353404 10.1016/s1470-2045(05)70458-7

[37] Rowland ST, Lebel ED, Goldman JSW, Domen JK, Bilsback KR, Ruiz A, et al. Downstream natural gas composition across U.S. and Canada: implications for indoor methane leaks and hazardous air pollutant exposures. Environ Res Lett. 2024;19:064064.

[38] US EPA. 2017 National Emissions Inventory (NEI) Data. 2021. https://www.epa.gov/air-emissions-inventories/2017-national-emissions-inventory-nei-data#datas (accessed 4 Mar2026).

