# Supplementary material for: Toxic Air Contaminant Emissions from Residential and Commercial Fossil Gas Appliances: A Systematic Review
Source: Curr Environ Health Rep. 2026 Sep 16;13(1):42. doi: 10.1007/s40572-026-00562-6 (PMC13578121; doi:10.1007/s40572-026-00562-6)
Supplement: Supplementary file 1 [file 40572_2026_562_MOESM1_ESM.docx]

**Supplement**

**Table S1. Web of Science search terms**

| **Search component** | **Terms** |
| --- | --- |
| Fuel type | (“gas” OR “natural gas” OR “fossil gas” OR "LPG" OR "liquefied petroleum gas" OR "propane" OR "biogas" OR "biomethane" OR "renewable natural gas" OR "butane") |
| Appliance type/location | (“appliance*” OR “stove*” OR “cooktop*” OR “gas range*” OR “hob*” OR “furnace*” OR “space heater*” OR “water heater*” OR “clothes dryer*” OR “fireplace*”) AND (“residential” OR “commercial” OR "household*" OR "outdoor*" OR "indoor*") |
| Emission-related metric | “emission*” OR “rate*” OR “emission factor*” OR “combustion” OR “leak*” OR “efficiency” |
| Pollutant (HAP, TAC, or UFPs) | “non-methane volatile organic compound*” OR “non methane volatile organic compound*” OR “NMVOC*” OR "VOC*" OR "volatile organic compound*" OR “benzene” OR “toluene” OR “ethylbenzene” OR “xylene*” OR "o-xylene" OR "ortho-xylene" OR "m-xylene" OR "meta-xylene" OR "p-xylene" OR "para-xylene" OR “BTEX” OR “formaldehyde” OR "HCHO" OR “hazardous air pollutant*” OR “HAP*” OR “toxic air contaminant*” OR “TAC*" OR "PM0.1" OR "ultrafine particulate*" OR "ultrafine particle*" OR "UFPs" OR "arsenic" OR "ethylene dibromide" OR "1,2-dibromoethane" OR "ethylene dichloride" OR "1,2-dichloroethane" OR "chromium" OR "asbestos" OR "dibenzo-p-dioxin*" OR "dibenzofuran*" OR "cadmium" OR "carbon tetrachloride" OR "tetrachloromethane" OR "ethylene oxide" OR "1,2-epoxyethane" OR "methylene chloride" OR "dichloromethane" OR "trichloroethylene" OR "trichloroethene" OR "chloroform" OR "vinyl chloride" OR "chloroethylene" OR "arsenic" OR "nickel" OR "perchloroethylene " OR "tetrachloroethylene" OR "1,3-butadiene" OR "lead" OR "acetaldehyde" OR "benzo[a]pyrene" OR "chlorinated dioxins" OR "chloroform |

**Section S1. Polycyclic Aromatic Hydrocarbons**

PAHs are commonly grouped by molecular weight (or number of aromatic rings) because these structural differences influence their formation during combustion, environmental behavior, and health relevance. Lower molecular weight PAHs (typically 2–3 rings) are generally more volatile and tend to partition into the gas phase, while higher molecular weight PAHs (≥4 rings) are less volatile and more likely to associate with particles. These differences affect how PAHs are emitted, transported, and measured, making molecular weight a useful framework for summarizing emission patterns.

Emission factors by compound group

EFs for PAHs were reported for LPG-fueled stove combustion across multiple studies ^27,33,34^. In total, forty-seven EF measurements were identified across individual PAH species and aggregated PAH groups. Reported EFs spanned 0.000035 to 0.04816 mg/MJ.

Lower molecular weight PAHs (e.g., naphthalene, methyl-naphthalenes, fluorene, acenaphthene, acenaphthylene) generally exhibited EFs ranging from approximately 0.000046 to 0.04816 mg/MJ ^27,33,34^. Naphthalene showed the highest EF among individual compounds, with values up to 0.04816 mg/MJ ^27^.

Intermediate molecular weight PAHs (e.g., phenanthrene, anthracene, fluoranthene, pyrene) exhibited EFs ranging from approximately 0.000187 to 0.03142 mg/MJ across studies ^27,33,34^. Fluoranthene and pyrene were among the more abundant PAHs reported, with EFs reaching 0.02885 mg/MJ and 0.03142 mg/MJ, respectively ^27^.

Higher molecular weight PAHs (e.g., benz[a]anthracene, chrysene, benzo[a]pyrene, indeno[1,2,3-cd]pyrene, benzo[ghi]perylene, dibenzo[a,h]anthracene) were generally reported at lower EF levels, typically between 0.000035 and 0.01197 mg/MJ ^27,34^.

Aggregated PAH emission factors

One study reported aggregated PAH emission factors. Total PAH₁₆ and PAH₂₅ emission factors were 0.0175 mg/MJ and 0.0183 mg/MJ, respectively ^34^. These values fell within the upper portion of the distribution observed for individual PAH species.

Consistency across compounds with multiple measurements

Several PAH species were reported across more than one study, allowing limited comparison of emission factors for the same compound. For example, anthracene EFs ranged from 0.000187 to 0.001085 mg/MJ across two studies ^33,34^. Similarly, fluoranthene and pyrene showed broader variability across studies, with reported EFs spanning 0.00576 to 0.02885 mg/MJ and 0.00195 to 0.03142 mg/MJ, respectively ^27,33,34^.

Several naphthalene derivatives, including 1-methylnaphthalene, 2-methylnaphthalene, and 2,6-dimethylnaphthalene, were also reported in multiple studies, with EFs generally on the order of 10⁻⁴ to 10⁻³ mg/MJ ^33,34^. Although the number of overlapping measurements is limited, these comparisons suggest that while absolute values vary across studies, reported emission factors for the same PAH species generally fall within the same order of magnitude.

Emission factors by appliance and fuel type

All identified PAH EFs corresponded to LPG-fueled stoves, with no measurements identified for natural gas appliances or for other appliance types. As a result, comparisons across fuels or appliances were not possible. Instead, variation in emission factors primarily reflected differences among individual PAH species and study-specific measurement conditions ^27,33,34^.

**Section S2. Other TACs**

This category includes several volatile organic compounds (VOCs) and carbonyl compounds reported less frequently in the literature but still relevant as combustion byproducts. Reported compounds include aromatic hydrocarbons (e.g., toluene, xylenes, ethylbenzene), alkenes (e.g., propylene), oxygenated carbonyls (e.g., acetaldehyde, acrolein, propionaldehyde, crotonaldehyde), and several chlorinated hydrocarbons ^27,29,32^. These species differ in volatility, atmospheric reactivity, and toxicity, but are commonly grouped together in emission inventories as individual VOC toxic air contaminants produced during incomplete combustion.

Emission factors by fuel type

EFs were reported for both natural gas and LPG appliances. LPG measurements were primarily derived from stove combustion experiments ^27,29^, while natural gas measurements included both stoves and dryers ^29,32^.

Across compounds, reported EFs spanned 1.2 × 10⁻⁴ to 3.064 mg/MJ. The highest emission factors were reported for acetaldehyde, with LPG stove measurements of 0.6428–3.064 mg/MJ ^27,29^. Other carbonyl compounds, including propionaldehyde, crotonaldehyde, and acrolein, exhibited LPG stove EFs generally between 0.0138 and 1.314 mg/MJ ^27,29^.

Aromatic VOCs such as toluene, ethylbenzene, and xylenes were reported at lower levels, typically ranging from 0.00094 to 0.085 mg/MJ across fuels and appliance types ^27,32^. Additional compounds including propylene, cyclohexane, and several chlorinated hydrocarbons were reported at similarly low EF levels ^27,32^.

Emission factors by appliance type

Most measurements corresponded to stove combustion experiments, particularly for LPG appliances ^27,29^. A smaller set of measurements corresponded to natural gas dryers, which reported emissions of aromatic hydrocarbons and chlorinated compounds including m,p-xylene, toluene, ethylbenzene, chloromethane, chloroethane, and carbon tetrachloride ^32^. Dryer EFs ranged from 1.2 × 10⁻⁴ to 0.085 mg/MJ, falling within the lower portion of the overall distribution of reported values.

Emission factors by fuel and appliance type

Stratification by both fuel type and appliance showed that LPG measurements were limited to stoves, while natural gas measurements included both stoves and dryers ^27,29,32^.

For LPG stoves, reported EFs spanned 0.00094 to 3.064 mg/MJ across a range of compound classes including carbonyls (e.g., acetaldehyde, acrolein, propionaldehyde, crotonaldehyde), aromatic hydrocarbons (e.g., toluene, ethylbenzene, xylenes), and cyclic hydrocarbons ^27,29^. Carbonyl compounds generally exhibited the highest EFs within this group, particularly acetaldehyde and crotonaldehyde.

For natural gas stoves, a smaller set of carbonyl compounds was reported, including acetaldehyde, propionaldehyde, and crotonaldehyde, with EFs ranging from 0.176 to 0.652 mg/MJ ^29^. These values fell within the broader distribution observed for LPG stove measurements but were generally lower than the highest LPG estimates.

For natural gas dryers, emissions were primarily associated with aromatic hydrocarbons and halogenated compounds, including toluene, ethylbenzene, m,p-xylene, chloromethane, chloroethane, and carbon tetrachloride, with EFs ranging from 1.2 × 10⁻⁴ to 0.085 mg/MJ ^32^. These values were typically lower than those observed for carbonyl compounds emitted from stoves.

Across fuel–appliance combinations, the highest emission factors were observed for LPG stove emissions of carbonyl compounds, while natural gas dryer emissions were generally an order of magnitude lower. However, the limited number of studies and differences in compounds measured across appliance types constrain direct comparisons between fuel–appliance categories.

Compounds with multiple measurements

Several compounds were reported in more than one study, allowing limited comparison across experiments. For example, acetaldehyde was measured in two LPG stove studies with EFs ranging from 0.6428 to 3.064 mg/MJ ^27,29^, indicating substantial variability across experiments. Propionaldehyde and crotonaldehyde were also reported in multiple measurements, with LPG stove EFs ranging from 0.0836 to 0.894 mg/MJ and 0.176 to 1.314 mg/MJ, respectively ^27,29^.

Similarly, ethylbenzene and m,p-xylene were reported for both LPG stoves and natural gas dryers, with EFs generally on the order of 10⁻³ to 10⁻² mg/MJ ^27,32^. Although the number of overlapping measurements remains limited, reported emission factors for the same compound generally fell within the same order of magnitude across studies.

**Section S3. Inferring TAC Emissions from Natural Gas Leakage**

Natural gas distributed to end users including residential and commercial end users can contain low but measurable molar ratios of certain TAC compounds such as BTEX compounds, hexane and cyclohexane ^23^. These compounds are present in trace quantities, originating in the geologic formation rock as a mixture alongside other hydrocarbons and impurities. While certain gas processing and pressure changes remove a significant portion of trace impurities, variable amounts can remain entrained throughout the supply chain and at the delivery point for residential and commercial end users. TAC compounds that remain entrained in natural gas at the point of consumption therefore can contribute to TAC emissions in the event of routine pneumatic leakage and routine pulses of unburned gas associated with normal operations (e.g., pulses during on/off cycling).

For example, measurements by Lebel et al. ^23^ showed benzene concentrations in whole, unburned natural gas throughout California pipeline end use systems (i.e., samples collected from gas stoves) ranging from 0.7–12 ppmv (max: 66 ppmv). Applying previously published behind-the-meter methane leakage estimates, Lebel et al., ^23^ estimated that 310 (95% CI: 140−980) kg benzene yr–1 is emitted in California from whole-house residential leaks, with an estimated 63 (95% CI: 32−150) kg benzene per year emitted from gas stove steady-state-off leakage alone. For comparison, the 2017 NEI estimated benzene emissions from residential NG combustion (not unburned leakage) in California to be 381 kg benzene yr–1 ^37^. This suggests that benzene emissions from building-level piping and appliances are equivalent to post-combustion emissions in addition to other aromatic compounds measured in pre-combusted fossil gas. Notably, Lebel et al., ^23^ did observe significant variability in BTEX content in fossil gas throughout California, likely reflective of the various sources of natural gas that are produced and imported to the consumption regions measured.

To infer TAC emissions from a natural gas sourced-leak that is primarily methane (>90%), a molar ratio of a TAC measured in unburned natural gas to methane measured in unburned natural gas can be assumed to be conserved in an open path leak at typical operating pressures of residential gas systems (e.g., 20 mbar gauge pressure). To formally scale measured methane (CH_4_) leakage rates by the molar fraction of a TAC of interest and identified in the gas stream, the following equation can be applied:

Where *E*_CH4_ is the methane emission rate [mass/time]; *C_TAC,_ C_CH4_* are mole fractions of the TAC and methane, respectively; and, MW_TAC,_ MW_CH4_ are molecular weights [g/mol]. Literature-derived methane EFs for stoves, ovens, and water heaters ^6,22,38^ can thus be used to approximate TAC emissions by multiplying with observed mole fractions of individual contaminants such as benzene.

The most comprehensive and contemporary database of constituents of natural gas for California is available from Lebel et al., ^23^. Table S1 from Lebel et al., ^23^ shows that seven TACs are commonly present in natural gas in California at the point of the residential appliance. These include BTEX, hexane, and cyclohexane that were detected in >97% of total samples collected and spanned a wide geographic extent. To accompany this dataset and apply inferred TAC emissions through leakage, this literature review also identified methane leakage studies that met the full inclusion criteria, except emission factors were for methane instead of a TAC. This literature review identified 14 methane studies that met this inclusion criteria and are included in a separate tab in the accompanying datasheet. However, it was beyond the scope of this review to formally calculate inferred TAC emissions based on these data sources.

**Section S4. Inferring TAC Emissions from Incomplete Combustion**

TAC emissions can be generated through incomplete combustion of fossil gas which occurs when oxygen availability is limited (i.e., fuel-rich) or flame conditions prevent full oxidation. Under such conditions, partial oxidation products such as carbon monoxide (CO), formaldehyde, and aromatic hydrocarbons, including benzene and PAHs, can form. While TAC production through this pathway is considered a secondary process that relies on multiple factors, a hallmark of incomplete combustion is the presence of CO. Unlike carbon dioxide, which is a marker of complete combustion or oxidation, the production of CO signals fuel-rich conditions that can result in partial oxidation and formation of TACs including formaldehyde, PAHs, benzene and others.

While methodologically intriguing, unfortunately this literature review identified only three studies between 1992 and 2023 that 1) simultaneously measured TAC EFs in addition to CO and 2) calculated or commented on the correlation between the measured TAC EFs and CO. Although several papers qualitatively noted that higher incomplete combustion or reduced air supply increased both CO and hydrocarbon emissions, few reported quantitative correlation coefficients between TAC EFs and CO emissions. This was mostly due to differences in study objectives and measurement methods for combustion gases vs TACs. The absence of such data limits the ability to use CO to infer TAC emissions; however, one such study is highlighted below to demonstrate both the strong relationship observed between CO and benzene as well as the method to infer TAC emissions using CO emissions only.

Kashtan et al. ^7^ analyzed 80 natural gas and propane burners and ovens throughout California and reported a robust and statistically significant correlation between benzene and CO emissions (R² = 0.67, p < 0.01). Based on their Figure S4, the slope of the relationship is approximately 0.18 µmol C_6_H_6_ per mmol CO. When converted to mass units:

Thus, an inferred benzene emission rate from CO measured from from residential gas cooking appliances can be written as:

To apply this statistical-based inferential equation elsewhere, suppose an emission rate of 100 mmol CO/hr was measured from a gas stove. Using the observed relationship between CO and benzene in Kashtan et al., ^7^, an inferred benzene emission rate of 1.41 mg/hr could be assumed. However, some caution or uncertainty bounds should be applied, as these data reflect data collected from a single study of 80 individual burner units. These values may be extrapolated to other non-stove appliances only if it can be sufficiently assumed that there is similarity in fuel chemistry and combustion dynamics. Unfortunately, no other non-stove studies were identified in this literature review that measured both CO and another TAC, limiting the confidence of such a generalization across appliance types. In practice, extrapolation must account for flame temperature, burner design, air-to-fuel ratios, and appliance load conditions which can vary by appliance type. Formation rates of certain TACs may also depend on local flame conditions not captured by CO alone.

Despite these limitations, evidence of TAC emissions exists for both CH_4_-based inference for leakage and CO-based inference for incomplete combustion. Additional empirical measurements are needed to both validate inference estimates particularly for the leakage pathways, and to provide additional statistical relationships between CO and other TACs particularly for non-stove appliances.
