# Supplementary material for: Toxic Air Contaminant Emissions from Residential and Commercial Fossil Gas Appliances: A Systematic Review
Source: Curr Environ Health Rep. 2026 Sep 16;13(1):42. doi: 10.1007/s40572-026-00562-6 (PMC13578121; doi:10.1007/s40572-026-00562-6)
Supplement: Supplementary file 2 [file 40572_2026_562_MOESM2_ESM.docx]

**Supplement**

**Table S1. Web of Science search terms**

| **Search component** | **Terms** |
| --- | --- |
| Fuel type | (“gas” OR “natural gas” OR “fossil gas” OR "LPG" OR "liquefied petroleum gas" OR "propane" OR "biogas" OR "biomethane" OR "renewable natural gas" OR "butane") |
| Appliance type/location | (“appliance*” OR “stove*” OR “cooktop*” OR “gas range*” OR “hob*” OR “furnace*” OR “space heater*” OR “water heater*” OR “clothes dryer*” OR “fireplace*”) AND (“residential” OR “commercial” OR "household*" OR "outdoor*" OR "indoor*") |
| Emission-related metric | “emission*” OR “rate*” OR “emission factor*” OR “combustion” OR “leak*” OR “efficiency” |
| Pollutant (HAP, TAC, or UFPs) | “non-methane volatile organic compound*” OR “non methane volatile organic compound*” OR “NMVOC*” OR "VOC*" OR "volatile organic compound*" OR “benzene” OR “toluene” OR “ethylbenzene” OR “xylene*” OR "o-xylene" OR "ortho-xylene" OR "m-xylene" OR "meta-xylene" OR "p-xylene" OR "para-xylene" OR “BTEX” OR “formaldehyde” OR "HCHO" OR “hazardous air pollutant*” OR “HAP*” OR “toxic air contaminant*” OR “TAC*" OR "PM0.1" OR "ultrafine particulate*" OR "ultrafine particle*" OR "UFPs" OR "arsenic" OR "ethylene dibromide" OR "1,2-dibromoethane" OR "ethylene dichloride" OR "1,2-dichloroethane" OR "chromium" OR "asbestos" OR "dibenzo-p-dioxin*" OR "dibenzofuran*" OR "cadmium" OR "carbon tetrachloride" OR "tetrachloromethane" OR "ethylene oxide" OR "1,2-epoxyethane" OR "methylene chloride" OR "dichloromethane" OR "trichloroethylene" OR "trichloroethene" OR "chloroform" OR "vinyl chloride" OR "chloroethylene" OR "arsenic" OR "nickel" OR "perchloroethylene " OR "tetrachloroethylene" OR "1,3-butadiene" OR "lead" OR "acetaldehyde" OR "benzo[a]pyrene" OR "chlorinated dioxins" OR "chloroform |

**Section S1. Polycyclic Aromatic Hydrocarbons**

PAHs are commonly grouped by molecular weight (or number of aromatic rings) because these structural differences influence their formation during combustion, environmental behavior, and health relevance. Lower molecular weight PAHs (typically 2–3 rings) are generally more volatile and tend to partition into the gas phase, while higher molecular weight PAHs (≥4 rings) are less volatile and more likely to associate with particles. These differences affect how PAHs are emitted, transported, and measured, making molecular weight a useful framework for summarizing emission patterns.

Emission factors by compound group

EFs for PAHs were reported primarily for LPG-fueled stove combustion across three studies (Bilsback et al.; Champion et al.; Shen et al.), with additional measurements for a natural gas heater reported by Lindberg et al. In total, 52 numeric EF measurements were identified across individual PAH species and aggregated PAH groups, with four additional measurements reported as below the detection limit (LDL). Reported EFs spanned 0.000035 to 0.04816 mg/MJ.

Lower molecular weight PAHs (e.g., naphthalene, methyl-naphthalenes, fluorene, acenaphthene, acenaphthylene) generally exhibited EFs ranging from approximately 0.000046 to 0.04816 mg/MJ for LPG stoves (Bilsback et al.; Champion et al.; Shen et al.). Natural gas heater measurements for naphthalene, 1-methylnaphthalene, 2-methylnaphthalene, and acenaphthylene ranged from 0.0000743 to 0.000209 mg/MJ (Lindberg et al.). Naphthalene showed the highest EF among individual compounds, with values up to 0.04816 mg/MJ for LPG stoves (Bilsback et al.).

Intermediate molecular weight PAHs (e.g., phenanthrene, anthracene, fluoranthene, pyrene) exhibited EFs ranging from approximately 0.000187 to 0.03142 mg/MJ across studies (Bilsback et al.; Champion et al.; Shen et al.). Fluoranthene and pyrene were among the more abundant PAHs reported, with EFs reaching 0.02885 mg/MJ and 0.03142 mg/MJ, respectively (Bilsback et al.).

Higher molecular weight PAHs (e.g., benz[a]anthracene, chrysene, benzo[a]pyrene, indeno[1,2,3-cd]pyrene, benzo[ghi]perylene, dibenzo[a,h]anthracene) were generally reported at lower EF levels, typically between 0.000035 and 0.01197 mg/MJ (Bilsback et al.; Shen et al.).

Aggregated PAH emission factors

One study reported aggregated PAH emission factors. Total PAH₁₆ and PAH₂₅ emission factors were 0.0175 mg/MJ and 0.0183 mg/MJ, respectively (Shen et al.). These values fell within the upper portion of the distribution observed for individual PAH species.

Consistency across compounds with multiple measurements

Several PAH species were reported across more than one study, allowing limited comparison of emission factors for the same compound. For example, anthracene EFs ranged from 0.000187 to 0.001085 mg/MJ across two studies (Champion et al.; Shen et al.). Similarly, fluoranthene and pyrene showed broader variability across studies, with reported EFs spanning 0.00576 to 0.02885 mg/MJ and 0.00195 to 0.03142 mg/MJ, respectively (Bilsback et al.; Champion et al.; Shen et al.).

Several naphthalene derivatives, including 1-methylnaphthalene and 2-methylnaphthalene, were reported across multiple studies and appliance/fuel combinations, with EFs generally on the order of 10⁻⁵ to 10⁻³ mg/MJ. Naphthalene and acenaphthylene were also reported across both LPG stoves and natural gas heaters. Although the number of overlapping measurements was limited, EFs for the same PAH species generally fell within a similar order of magnitude, with some variability across studies and combustion conditions.

Emission factors by appliance and fuel type

The available PAH evidence was concentrated primarily among LPG-fueled stoves, which accounted for the large majority of reported EFs and included compounds spanning lower to higher molecular weights. A smaller number of EFs were available for natural gas heaters, limited to lower molecular weight PAHs including naphthalene, 1-methylnaphthalene, 2-methylnaphthalene, and acenaphthylene. These natural gas heater EFs generally fell toward the lower end of the ranges reported for the corresponding compounds from LPG stoves. However, given the small number of natural gas measurements and differences in appliance type and study conditions, the available evidence does not support direct comparisons of PAH emissions by fuel or appliance type.

**Section S2. Other TACs**

This category includes several volatile organic compounds (VOCs) and carbonyl compounds reported less frequently in the literature but still relevant as combustion byproducts. Reported compounds include aromatic hydrocarbons (e.g., toluene, xylenes, ethylbenzene), alkenes (e.g., propylene), alkanes (e.g., n-hexane), oxygenated carbonyls (e.g., acetaldehyde, acrolein, propionaldehyde, crotonaldehyde), and several chlorinated hydrocarbons and other halogenated combustion products (Bilsback et al.; Van Winkle and Scheff; Zhang and Smith; Pfeiffer; Lindberg et al.). These species differ in volatility, atmospheric reactivity, and toxicity and represent a heterogeneous group of less frequently characterized TACs emitted from gas appliances.

Emission factors by fuel type

EFs were reported for both natural gas and LPG appliances. LPG measurements were derived from stove combustion experiments (Bilsback et al.; Zhang and Smith), while natural gas measurements spanned stoves, dryers, and heaters (Lindberg et al.; Pfeiffer; Van Winkle and Scheff; Zhang and Smith).

Across compounds, reported EFs spanned several orders of magnitude, from 1.67 × 10⁻⁶ mg I-TEQ/MJ for PCDD/PCDF to 3.064 mg/MJ for acetaldehyde, although the toxicity-equivalent units used for PCDD/PCDF are not directly comparable with the mass-based EFs reported for other compounds. Among EFs expressed as compound mass per unit energy, values ranged from 1.2 × 10⁻⁴ to 3.064 mg/MJ. The highest emission factors were reported for acetaldehyde, with LPG stove measurements of 0.6428–3.064 mg/MJ (Bilsback et al.; Zhang and Smith). Other carbonyl compounds, including propionaldehyde, crotonaldehyde, and acrolein, exhibited LPG stove EFs generally between 0.0138 and 1.314 mg/MJ (Bilsback et al.; Zhang and Smith).

Aromatic VOCs such as toluene, ethylbenzene, and xylenes were reported at lower levels, with quantifiable EFs ranging from 0.00094 to 0.0979 mg/MJ across fuels and appliance types (Bilsback et al.; Lindberg et al.; Van Winkle and Scheff). Additional compounds including propylene, cyclohexane, n-hexane, and several chlorinated hydrocarbons were reported at similarly low EF levels.

Emission factors by appliance type

Most measurements corresponded to stove combustion experiments, particularly for LPG appliances (Bilsback et al.; Zhang and Smith). Natural gas appliances were represented by a smaller number of measurements from stoves, dryers, and heaters. Natural gas dryers emitted aromatic hydrocarbons and chlorinated compounds including m,p-xylene, toluene, ethylbenzene, chloromethane, chloroethane, and carbon tetrachloride, as well as propylene and 1,1,2,2-tetrachloroethane (Van Winkle and Scheff). Quantifiable dryer EFs ranged from 1.2 × 10⁻⁴ to 0.085 mg/MJ.

Natural gas heater measurements included toluene and n-hexane, with EFs of 0.0979 and 0.00712 mg/MJ, respectively (Lindberg et al.). PCDD/PCDF emissions were also reported for a natural gas heater at 1.67 × 10⁻⁶ mg I-TEQ/MJ (Pfeiffer). Overall, however, the evidence for heaters and dryers was considerably more limited than that for stoves and covered different sets of compounds, limiting direct comparisons among appliance types.

Emission factors by fuel and appliance type

Stratification by both fuel type and appliance showed that LPG measurements were limited to stoves, while natural gas measurements included stoves, dryers, and heaters (Bilsback et al.; Lindberg et al.; Pfeiffer; Van Winkle and Scheff; Zhang and Smith).

For LPG stoves, reported EFs spanned 0.00094 to 3.064 mg/MJ across a range of compound classes including carbonyls (e.g., acetaldehyde, acrolein, propionaldehyde, crotonaldehyde), aromatic hydrocarbons (e.g., toluene, ethylbenzene, xylenes), and cyclic hydrocarbons (Bilsback et al.; Zhang and Smith). Carbonyl compounds generally exhibited the highest EFs within this group, particularly acetaldehyde and crotonaldehyde.

For natural gas stoves, a smaller set of carbonyl compounds was reported, including acetaldehyde, propionaldehyde, and crotonaldehyde, with EFs ranging from 0.176 to 0.652 mg/MJ (Zhang and Smith). These values fell within the broader distribution observed for LPG stove measurements but were generally lower than the highest LPG estimates.

For natural gas dryers, emissions included aromatic hydrocarbons, halogenated compounds, and propylene, with quantifiable EFs ranging from 1.2 × 10⁻⁴ to 0.085 mg/MJ (Van Winkle and Scheff). Natural gas heater measurements covered a more limited set of compounds, including toluene (0.0979 mg/MJ), n-hexane (0.00712 mg/MJ), and PCDD/PCDF (1.67 × 10⁻⁶ mg I-TEQ/MJ) (Lindberg et al.; Pfeiffer.).

Across fuel–appliance combinations, the largest mass-based EFs were reported for carbonyl compounds from LPG stoves. However, the evidence base was unevenly distributed across appliance categories: stove studies characterized a broader range of carbonyls and VOCs, whereas dryer and heater studies measured smaller and largely different sets of compounds. Consequently, differences in observed EF distributions across appliance categories may reflect both true differences in emissions and differences in the compounds targeted by individual studies, limiting direct comparisons across fuels and appliances.

Compounds with multiple measurements

Several compounds were reported in more than one study or fuel–appliance combination, allowing limited comparison across experiments. For example, acetaldehyde was measured in two LPG stove studies with EFs ranging from 0.6428 to 3.064 mg/MJ (Bilsback et al.; Zhang and Smith), indicating substantial variability across experiments. Propionaldehyde and crotonaldehyde were also reported in multiple measurements, with LPG stove EFs ranging from 0.0836 to 0.894 mg/MJ and 0.176 to 1.314 mg/MJ, respectively (Bilsback et al.; Zhang and Smith).

Several aromatic VOCs were measured across different fuel–appliance combinations. Ethylbenzene and m,p-xylene were reported for both LPG stoves and natural gas dryers, while toluene was reported for LPG stoves, natural gas dryers, and natural gas heaters. Toluene EFs ranged from 0.04383 mg/MJ for an LPG stove to 0.0979 mg/MJ for a natural gas heater, with the natural gas dryer measurement falling between these values at 0.070 mg/MJ. Although these overlapping measurements provide some indication of the range of EFs for individual compounds, the small number of studies and differences in appliance and experimental conditions preclude robust comparisons across fuel–appliance combinations.

**Section S3. Inferring TAC Emissions from Natural Gas Leakage**

Natural gas distributed to end users including residential and commercial end users can contain low but measurable molar ratios of certain TAC compounds such as BTEX compounds, hexane and cyclohexane (Lebel, Michanowicz, et al.). These compounds are present in trace quantities, originating in the geologic formation rock as a mixture alongside other hydrocarbons and impurities. While certain gas processing and pressure changes remove a significant portion of trace impurities, variable amounts can remain entrained throughout the supply chain and at the delivery point for residential and commercial end users. TAC compounds that remain entrained in natural gas at the point of consumption therefore can contribute to TAC emissions in the event of routine pneumatic leakage and routine pulses of unburned gas associated with normal operations (e.g., pulses during on/off cycling).

For example, measurements by Lebel and colleagues showed benzene concentrations in whole, unburned natural gas throughout California pipeline end use systems (i.e., samples collected from gas stoves) ranging from 0.7–12 ppmv (max: 66 ppmv) (Lebel, Michanowicz, et al.). Applying previously published behind-the-meter methane leakage estimates, Lebel et al. estimated that 310 (95% CI: 140−980) kg benzene yr–1 is emitted in California from whole-house residential leaks, with an estimated 63 (95% CI: 32−150) kg benzene per year emitted from gas stove steady-state-off leakage alone. For comparison, the 2017 NEI estimated benzene emissions from residential NG combustion (not unburned leakage) in California to be 381 kg benzene yr–1 (US EPA). This suggests that benzene emissions from building-level piping and appliances are equivalent to post-combustion emissions in addition to other aromatic compounds measured in pre-combusted fossil gas. Notably, Lebel et al. did observe significant variability in BTEX content in fossil gas throughout California, likely reflective of the various sources of natural gas that are produced and imported to the consumption regions measured (Lebel, Michanowicz, et al.).

To infer TAC emissions from a natural gas sourced-leak that is primarily methane (>90%), a molar ratio of a TAC measured in unburned natural gas to methane measured in unburned natural gas can be assumed to be conserved in an open path leak at typical operating pressures of residential gas systems (e.g., 20 mbar gauge pressure). To formally scale measured methane (CH_4_) leakage rates by the molar fraction of a TAC of interest and identified in the gas stream, the following equation can be applied:

Where *E*_CH4_ is the methane emission rate [mass/time]; *C_TAC,_ C_CH4_* are mole fractions of the TAC and methane, respectively; and, MW_TAC,_ MW_CH4_ are molecular weights [g/mol]. Literature-derived methane EFs for stoves, ovens, and water heaters (Fischer et al.; Lebel, Finnegan, et al.; Nicholas et al.) can thus be used to approximate TAC emissions by multiplying with observed mole fractions of individual contaminants such as benzene.

Lebel and colleagues developed the most comprehensive and contemporary database of natural gas constituents in California (Lebel, Michanowicz, et al.). Table S1 shows that seven TACs are commonly present in natural gas in California at the point of the residential appliance (Lebel, Michanowicz, et al.). These include BTEX, hexane, and cyclohexane that were detected in >97% of total samples collected and spanned a wide geographic extent. To accompany this dataset and apply inferred TAC emissions through leakage, this literature review also identified methane leakage studies that met the full inclusion criteria, except emission factors were for methane instead of a TAC. This literature review identified 14 methane studies that met this inclusion criteria and are included in a separate tab in the accompanying datasheet. However, it was beyond the scope of this review to formally calculate inferred TAC emissions based on these data sources.

**Section S4. Inferring TAC Emissions from Incomplete Combustion**

TAC emissions can be generated through incomplete combustion of fossil gas which occurs when oxygen availability is limited (i.e., fuel-rich) or flame conditions prevent full oxidation. Under such conditions, partial oxidation products such as carbon monoxide (CO), formaldehyde, and aromatic hydrocarbons, including benzene and PAHs, can form. While TAC production through this pathway is considered a secondary process that relies on multiple factors, a hallmark of incomplete combustion is the presence of CO. Unlike carbon dioxide, which is a marker of complete combustion or oxidation, the production of CO signals fuel-rich conditions that can result in partial oxidation and formation of TACs including formaldehyde, PAHs, benzene and others.

While methodologically intriguing, unfortunately this literature review identified only three studies between 1992 and 2023 that 1) simultaneously measured TAC EFs in addition to CO and 2) calculated or commented on the correlation between the measured TAC EFs and CO. Although several papers qualitatively noted that higher incomplete combustion or reduced air supply increased both CO and hydrocarbon emissions, few reported quantitative correlation coefficients between TAC EFs and CO emissions. This was mostly due to differences in study objectives and measurement methods for combustion gases vs TACs. The absence of such data limits the ability to use CO to infer TAC emissions; however, one such study is highlighted below to demonstrate both the strong relationship observed between CO and benzene as well as the method to infer TAC emissions using CO emissions only.

Kashtan and colleagues analyzed 80 natural gas and propane burners and ovens throughout California and reported a robust and statistically significant correlation between benzene and CO emissions (R² = 0.67, p < 0.01) (Kashtan et al.). Based on their Figure S4, the slope of the relationship is approximately 0.18 µmol C_6_H_6_ per mmol CO. When converted to mass units:

Thus, an inferred benzene emission rate from CO measured from from residential gas cooking appliances can be written as:

To apply this statistical-based inferential equation elsewhere, suppose an emission rate of 100 mmol CO/hr was measured from a gas stove. Using the observed relationship between CO and benzene by Kashtan and colleagues, an inferred benzene emission rate of 1.41 mg/hr could be assumed (Kashtan et al.). However, some caution or uncertainty bounds should be applied, as these data reflect data collected from a single study of 80 individual burner units. These values may be extrapolated to other non-stove appliances only if it can be sufficiently assumed that there is similarity in fuel chemistry and combustion dynamics. Unfortunately, no other non-stove studies were identified in this literature review that measured both CO and another TAC, limiting the confidence of such a generalization across appliance types. In practice, extrapolation must account for flame temperature, burner design, air-to-fuel ratios, and appliance load conditions which can vary by appliance type. Formation rates of certain TACs may also depend on local flame conditions not captured by CO alone.

Despite these limitations, evidence of TAC emissions exists for both CH_4_-based inference for leakage and CO-based inference for incomplete combustion. Additional empirical measurements are needed to both validate inference estimates particularly for the leakage pathways, and to provide additional statistical relationships between CO and other TACs particularly for non-stove appliances.
